# Application of Jiawei Maxing Shigan Tang in the treatment of COVID-19: An observational study

**DOI:** 10.3389/fmed.2022.1028171

**Published:** 2022-10-20

**Authors:** Jia Wu, Feng Tang, Xiao-Qiang Zhang, Zai-Lin Fu, Shui Fu

**Affiliations:** ^1^Department of Pharmacy, Linping Campus, The Second Affiliated Hospital of Zhejiang University School of Medicine, Hangzhou, China; ^2^Department of Traditional Chinese Medicine, Linping Campus, The Second Affiliated Hospital of Zhejiang University School of Medicine, Hangzhou, China; ^3^Department of Infection, Linping Campus, The Second Affiliated Hospital of Zhejiang University School of Medicine, Hangzhou, China; ^4^Department of Clinical Laboratory, Linping Campus, The Second Affiliated Hospital of Zhejiang University School of Medicine, Hangzhou, China

**Keywords:** Jiawei Maxing Shigan Tang, COVID-19, efficacy comparison, adverse reactions, lung imaging, lymphocyte

## Abstract

**Objective:**

To explore the clinical efficacy and adverse reactions of Jiawei Maxing Shigan Tang (JMST; a modified decoction of ephedra, apricot kernel, gypsum, and licorice) combined with western medicine in the symptomatic treatment of COVID-19.

**Methods:**

In this study, we retrospectively collected the basic data of 48 patients with COVID-19 who were discharged from our hospital between January 20 and February 28, 2020. Besides, the blood routines, biochemical indexes, nucleic acid detection results, clinical symptoms, lung imaging improvements, adverse reactions, and other clinical data of these patients before and after treatment were recorded. Finally, we drew comparisons between the outcomes and adverse reactions of patients in the combined treatment group (therapeutic regimen recommended by authoritative guidelines and supplemented by JMST) and the conventional treatment group (therapeutic regimen recommended by authoritative guidelines).

**Results:**

There were no significant differences in age, gender, clinical classification, and underlying medical conditions between the combined treatment group (28 cases) and the conventional treatment group (20 cases). However, the combined treatment group presented superior results to the conventional treatment group in several key areas. For instance, patients produced negative nasal/throat swab-based nucleic acid detection results in a shorter time, clinical symptoms were more effectively alleviated, and the absorption time of lung exudation was shorter (*P* < 0.05). Furthermore, the combined treatment group had a shorter length of stay (LOS) and faster lymphocyte recovery duration than the conventional treatment group, although the differences were not statistically significant. Moreover, there were no significant differences concerning gastrointestinal reaction, hepatic injury, renal impairment, myocardial injury, and other adverse reactions between the two groups.

**Conclusion:**

The results of this study indicate that JMST combined with the recommended therapeutic regimen enhances the recovery of COVID-19 patients without increasing the risk of adverse reactions. Therefore, this therapy promotes positive outcomes for COVID-19 patients.

## Introduction

The coronavirus disease (COVID-19), caused by severe acute respiratory syndrome coronavirus 2(SARS-CoV-2), a highly pathogenic novel coronavirus, has started a worldwide pandemic since December 2019. COVID-19 has received a great deal of attention from all sectors of society due to its threat to the lives and health of people, economic development, and social stability worldwide (1, 2). As a result, several versions of the *Diagnosis and Treatment Protocol for COVID-19* have been published in China (3–5). According to the World Health Organization (WHO), COVID-19 has been designated “2019-nCoV acute respiratory disease”. The treatment of patients diagnosed with COVID-19 is an important component of epidemic prevention and control and it is also the primary measure to protect people's lives. To date, there are no specific drugs for treating this disease in clinical applications, but the efficacy of traditional Chinese medicine (TCM) in the treatment of severe acute respiratory syndrome (SARS) has been verified (6, 7). Because novel coronavirus and SARS-COV exhibit high homology in gene sequencing (8), it can be inferred that Chinese herbal medicine also has certain effects in the treatment of COVID-19. Thus, combined therapies that integrate TCM and western medicine may be promising treatment methods. Our hospital is designated to receive patients with COVID-19 and is a National General Hospital Traditional Chinese Medicine Work Demonstration Unit. The TCM experts in our hospital extracted the essence from ancient recipes and applied them to the diagnosis and treatment protocols for COVID-19. They rapidly formulated a TCM therapeutic regimen for COVID-19 patients by actively implementing the latest national diagnosis and treatment protocols for COVID-19 in combination with their experience in clinical diagnosis and treatment. Based on these efforts, patients with COVID-19 were treated promptly with TCM. In this study, 48 COVID-19 patients were treated with Jiawei Maxing Shigan Tang (JMST), a modified decoction of ephedra, apricot kernel, gypsum, and licorice, and an established prescription of our hospital. Their treatment was continuously optimized in clinical practice to assist experts in western medicine. We established that a combination of TCM and western medicine contributed to realizing a superposition effect. Furthermore, TCM promoted the recovery of patients at the convalescent stage, thereby achieving favorable clinical outcomes. Exploring the effectiveness of combined therapies integrating TCM and western medicine is expected to provide a valuable reference for clinicians who are engaged in the fight against the COVID-19 pandemic.

## Data and methods

### Participants

In this study, we retrospectively collected the data from a total of 48 patients with common COVID-19 who were discharged from our hospital between January 20 and February 28, 2020. The cohort included 26 males and 22 females with an average age of 47.54 ± 13.29 years, ranging from 29 to 70 years old. The diagnosis, classification, and medication of all participants were performed based on the *Diagnosis and Treatment Protocol for COVID-19 (Trial Version 5)* (5) and the *Recommended Scheme for Prevention and Treatment of COVID-19 by Traditional Chinese Medicine (Trial Version 4) in Zhejiang Province* (9). All participants included in the study received treatment based on the therapeutic regimen for at least 1 week. The inclusion criteria included: (1) patients with positive nucleic acid detection results in blood/respiratory tract samples by real-time PCR (RT-PCR); (2) patients with signs of viral pneumonia, such as ground glass opacity (GGO) and mixed ground glass opacity (mGGO), in a spiral CT scan of the chest; (3) patients who had signed informed consent. The exclusion criteria were: (1) pregnant patients; (2) pediatric patients; (3) patients with ongoing immunosuppressive therapy; (4) patients with acquired immunodeficiency syndrome (AIDS); (5) patients who recovered within 48 h. The criteria for release from isolation and discharge were: (1) patients whose clinical symptoms (high body temperature, cough, expectoration, physical strength, and other related symptoms) were alleviated or had disappeared; (2) patients with more than two negative nucleic acid detection results from respiratory tract samples, with sampling performed over at least 2 days; (3) patients with negative nucleic acid detection results of fecal samples. Those who met all of the above three criteria were considered cured and were discharged from the hospital.

### Treatment methods and groupings

The patients were divided into the conventional treatment group and the combined treatment group, which involved combining JMST and conventional treatment. The routine treatment group was administered: lopinavir/ritonavir (200 mg/50 mg per tablet), two tablets/time, twice a day; arbidol, 200 mg/time, three times a day; aerosol inhalation of interferon-α, 5 million U/time, twice a day. The JMST prescription consisted of 30 g gypsum (decocted later), 10 g Rhizoma atractylodis, 10 g notopterygium root, 10 g Agastache rugosa, 9 g apricot kernel, 9 g betel nut, 9 g dried tangerine peel, 6 g ephedra, 6 g licorice, and 6 g amomum tsao-ko. Among the cohort, there were 20 patients in the conventional treatment group, including 12 males and 8 females, with an age of 47.66 ± 13.56 years, ranging from 30 to 70 years old. The combined treatment group consisted of 28 subjects, including 16 males and 12 females, with an average age of 47.45 ± 13.21 years, ranging from 29 to 69 years old.

### Data collection

#### General data

General data included gender, age, duration from onset of symptoms to treatment, and underlying medical conditions. The patients were subjected to scientific clinical classification according to the *Diagnosis and Treatment Protocol for COVID-19 (Trial Version 5)*, based on their symptoms, signs, lung imaging features, laboratory examination results, and treatment information at the time of admission.

#### Clinical efficacy

(1) Length of stay (LOS): The LOS was calculated from the date of confirmed diagnosis based on positive nucleic acid detection results to the date of discharge based on the discharge standard. (2) Remission duration of the main symptoms: This was determined as the time from the date of onset and appearance of symptoms to the date the clinical symptoms disappeared, according to medical records. Clinical symptoms included higher body temperature, cough, expectoration, and respiratory symptoms.

#### Imaging improvement

(1) Duration for the initial absorption of lung infection lesions: calculated as the time from the date of the first chest CT scan to that of initial improvement in acute exudative lesions. (2) Duration for the complete absorption of lung lesions: the number of days between the first chest CT scan and the complete elimination of acute exudative lesions.

#### Duration for nasal/throat swab-based nucleic acid detection results to become negative

This was calculated as the time from initially obtaining positive nucleic acid detection results to the date of obtaining negative nucleic acid detection results in the respiratory tract and fecal samples more than twice with a sampling interval of 1 day.

#### Lymphocyte recovery duration

According to the national treatment standard, we tested the levels of white blood cells, lymphocytes, ALT, and calcitonin. We discovered that only the lymphocyte count decreased in all patients enrolled in clinical laboratory index detection. Hence, the lymphocyte count was selected as the observation index. The lymphocyte recovery duration was expressed as the number of days from obtaining positive nucleic acid detection results to when the lymphocyte count returned to within the normal reference levels.

#### Adverse reactions

(1) Gastrointestinal symptoms: If at least one gastrointestinal symptom, such as abdominal distension, abdominal pain, nausea, vomiting, eructation, hiccupping, eating obstruction, or pain occurred in patients, the patients were diagnosed with gastrointestinal reactions; (2) Liver dysfunction: If the levels of one or more indexes, including serum alanine aminotransferase (ALT), aspartate aminotransferase (AST), total bilirubin (TBIL), or γ-glutamyltranspeptidase (γ-GT), significantly increased in patients, they were diagnosed with liver dysfunction; (3) Renal impairment: If one or more indexes, such as serum blood urea nitrogen (BUN), creatinine (Cr), and uric acid (UA) exhibited substantially increased levels in patients, they were diagnosed with renal impairment; (4) Myocardial injury: If any of the following indexes: serum creatine kinase isoenzyme-MB (CK-MB), lactate dehydrogenase (LDH), orcardiac troponin I (TnI) had significantly increased levels, the patients were diagnosed with myocardial injury.

### Statistical methods

In this study, SPSS 26.0 was utilized to process the data and the Shapiro-Wilk test was conducted to analyze data distribution. The data with normal distribution were expressed by $\bar{x}$ ± s. The independent sample *t*-test was performed to make comparisons between groups, and the chi-square (χ^2^) test was employed to compare the rates, Additionally, the enumeration data were analyzed using the Wilcoxon-Mann-Whitney rank sum test. Values of *P* < 0.05 indicated that there was a statistically significant difference.

## Results

### Basic data comparison of patients in both groups

In this study, we collected the basic data of 48 patients in two groups. As Table 1 indicates, there were no significant differences in gender distribution, age distribution, duration from onset of symptoms to treatment, clinical classification and underlying medical conditions between the groups (*P* > 0.05).

**Table 1:** General data analysis of patients in both groups.

**Group**	**Cases**	**Gender (cases/%)**		**Age** **(years, $\bar{x}$ ±s)**	**Clinical classification (case/%)**				**Duration from** **onset of symptoms** **to treatment** **(days, $\bar{x}$ ±s)**	**Basic diseases (cases/%)**				
		**Male**	**Female**		**Mild**	**Moderate**	**Severe**	**Critical**		**Diabetes** **mellitus**	**Hypertension**	**Lung** **disease**	**Tumor**	**No** **underlying** **medical** **conditions**
Conventional treatment group	20	12/60	8/40	47.66 ± 13.56	1/5	17/85	2/10	0/0	3.14 ± 0.76	1/5	5/25	3/15	2/10	9/45
Combined treatment group	28	16/57.14	12/42.86	47.45 ± 13.21	2/7.14	23/82.15	3/10.71	0/0	3.25 ± 0.81	2/7.14	9/32.14	6/21.43	3/10.71	8/28.58
Statistical value	—	χ2 = 0.039		t = 0.678	Z = −0.571				t = 0.932	Z = −1.056				
*P* value	—	0.843		0.532	0.568				0.216	0.291				

### LOS and remission duration of main symptoms

There was no significant difference in average LOS between the two groups. However, the time for clinical symptoms to disappear in the combined treatment group was much shorter than in the conventional treatment group, and the difference was statistically significant (Table 2).

**Table 2:** Comparison of length of stay and time for clinical symptoms to disappear between the groups.

**Group**	**Cases**	**Length of** **stay** **(days, $\bar{x}$ ±s)**	**Time for clinical** **symptoms to** **disappear** **(days, $\bar{x}$ ±s)**
Conventional treatment group	20	23.78 ± 4.48	18.25 ± 2.44
Combined treatment group	28	22.60 ± 3.56	12.14 ± 1.76
*t* value	—	0.328	2.387
*P* value	—	0.747	0.049

### Imaging improvement

The duration of initial and complete absorption/obvious absorption of lung exudation lesions in the combined treatment group was considerably shorter than in the conventional treatment group, with a statistically significant difference. However, the complete absorption rate in the combined treatment group was higher than that in the conventional treatment group, but the difference was not statistically significant (Table 3).

**Table 3:** Comparison of the initial and complete absorption duration of lung infection lesions between the groups.

**Group**	**Cases**	**Initial absorption** **duration (days, $\bar{x}$ ±s)**	**Obvious or complete** **absorption duration** **(days, $\bar{x}$ ±s)**	**Complete** **absorption cases** **(cases/%)**
Conventional treatment group	20	9.23 ± 1.21	22.78 ± 4.31	8/40
Combined treatment group	28	7.56 ± 1.02	13.09 ± 2.31	15/53.57
Statistical value	—	t = 1.956	t = 2.243	χ2 = 0.861
*P*	—	0.042	0.040	0.353

### Main laboratory indexes

**Figure 1:**
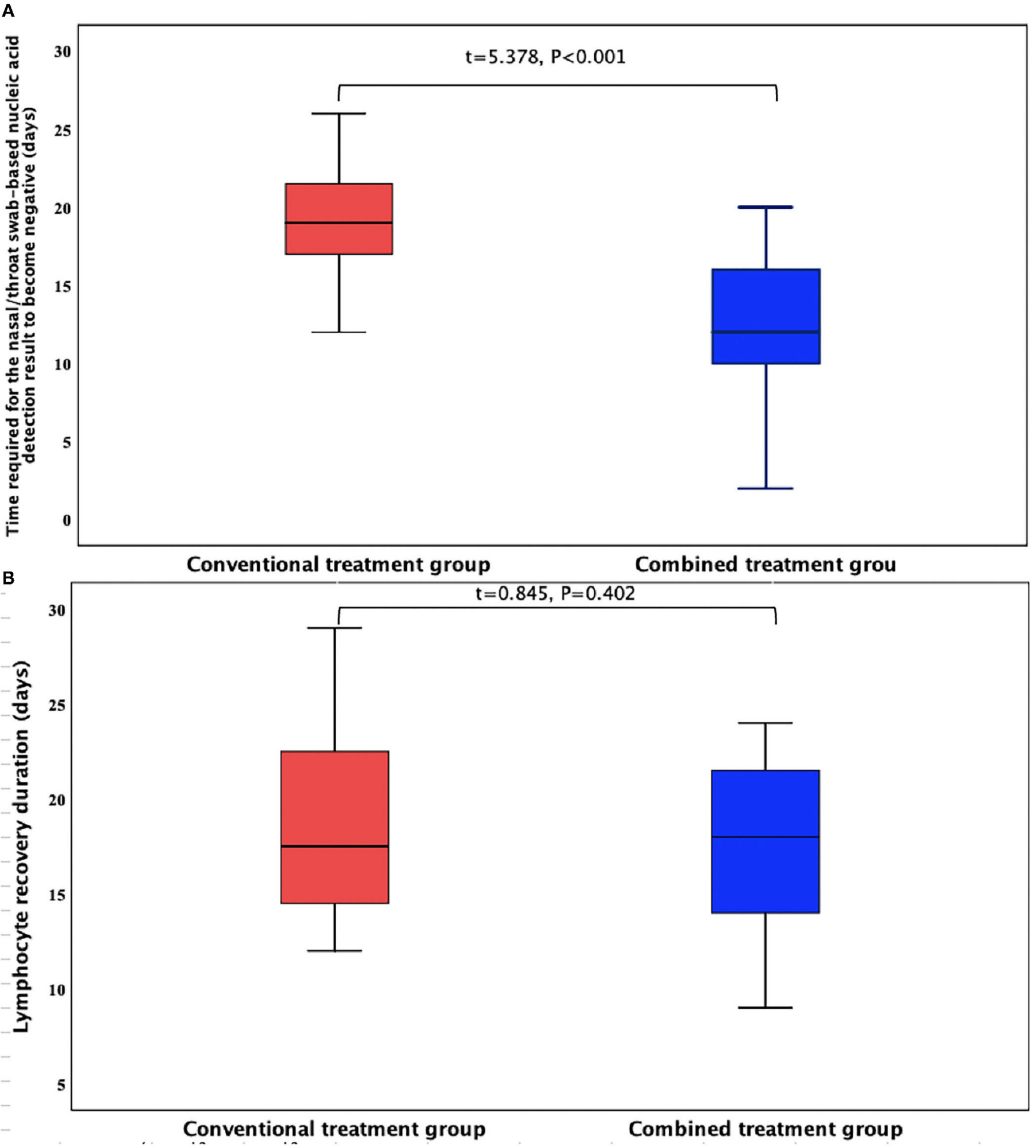
Comparison of the main experimental indexes of the two patient groups. **(A)** Time required for the nasal/throat swab-based nucleic acid detection result to become negative (days); **(B)** Lymphocyte recovery duration (days).

The duration required for the nasal/throat swab-based nucleic acid detection result to become negative was substantially shorter in the combined treatment group than in the conventional treatment group, and the difference was statistically significant. However, there was no significant difference in the lymphocyte recovery duration between the two groups (Figure 1).

### Adverse reactions

Gastrointestinal reaction, hepatic injury, renal impairment, and myocardial injury were slightly higher in the combined treatment group than in the conventional treatment group, but the difference was not statistically significant (Table 4).

**Table 4:** Incidence of adverse reactions in both groups (cases/%).

**Group**	**Cases**	**Gastrointestinal reaction**	**Hepatic injury**	**Renal impairment**	**Myocardial injury**
Conventional treatment group	20	7/35	5/25	3/15	1/5
Combined treatment group	28	13/46.43	9/32.14	7/25	2/7.15
χ^2^ value	—	0.627	0.288	0.231	0.091
*P* value	—	0.428	0.591	0.631	0.762

## Discussion

In recent years, there have been an increasing number of new viral infectious diseases. However, effective treatment methods have not been implemented for several of these illnesses, and acute outbreaks often cause panic and become major social problems. The effectiveness of several drugs in TCM and western medicine for the treatment of COVID-19 remains unclear. However, from the perspective of TCM, the regular forms of the body under the attack of foreign pathogens can be determined, even though COVID-19 is a new disease. TCM had a vital role in the treatment of SARS in 2003, so we can presume that TCM can also play a crucial part in the prophylaxis and treatment of COVID-19 (10, 11).

Hangzhou is located to the south of the Yangtze River, and the latitude of Hangzhou is approximately 30° N, which is the same as Wuhan. In the winter of 2019, the weather was abnormally cold and damp. Besides, rainy weather dominated from January to mid-February, and Wang et al. (10) also proposed the “wet” evil in their report. Additionally, Zhang et al. (12) proposed that the distribution of urban residents in Hangzhou with a moderate or biased constitution had similar proportions (52.6 vs. 47.4%).The 48 patients in this study had a history of living in Hubei Province or had come into contact with COVID-19 patients in Hubei Province or Hangzhou within 14 days of disease onset, with a specific etiology, location, and symptoms. According to *Wenyi Lun*, compiled by Wu Youke in the Qing Dynasty, “In years with a strong or weak abnormal climate, those who have a slight decline in healthy energy will get sick when they touch it”; “disease occurs either in the city or in the village, anywhere with a crowd”; “individuals from all families are equal when encountering disease”. In TCM, the term “plague” refers to a kind of disease with strong infectivity and the potential to cause epidemics. Therefore, COVID-19 belongs to a disease category within TCM that is caused by cold-damp epidemic pathogenic factors. Tong (13) also defined the TCM name of novel coronavirus pneumonia as “cold-damp disease”. COVID-19 is caused by damp-heat epidemic pathogenic factors. Hence, its core pathogenesis lies in dampness and toxins, combined with cold and heat. After the pathogenic factors affect the ying blood, the yang qi or yin fluids are damaged to varying degrees. The lungs and spleen are mainly involved, while the heart, liver, kidneys, and other organs are affected in severe cases.

According to *Su Wen – CifaLun* (14), five infectious diseases are prone to becoming pandemic, and there are similar symptoms among the different severity levels. Based on the *Diagnosis and Treatment Protocol for COVID-19* issued by the National Health Commission and National Administration of Traditional Chinese Medicine, our hospital focuses on treatments based on disease differentiation, according to epidemic features and similar symptoms. JMST is also widely adopted as a general treatment. “If the evil is not eliminated, the disease will not be cured; it will be aggravated due to delayed treatment over a long period.” The principle of JMST treatment is to dispel cold, remove dampness, avoid filth, and convert turbidity. As the main component of JMST, the recipe for Maxing Shigan Tang (MST) is derived from the traditional medical text *Shanghan Lun*. The original prescription is mainly used to treat taiyang diseases and is a basic treatment for asthma and cough with unsolved exogenous pathogens and evil heat constraints in the lung pattern. Combined with betel nut, amomum tsao-ko, and other herbs, which are the main components of the classic prescription Dayuan-Yin from *Wenyi Lun*, MST can be modified according to the appearance of the patient's tongue and other concurrent syndromes. In the decoction, ephedra spreads lung qi, relieves asthma, and disperses evil; apricot kernel lowers lung qi; gypsum relieves lung heat; amomum tsao-ko features a pungent flavor and suppresses evil, eliminates filth, and prevents vomiting; betel nut disperses damp evil, eliminates phlegm, breaks constraints, and eliminates evil; Rhizoma atractylodis eliminates dampness, invigorates the spleen, expels wind, and removes cold; notopterygium root generates warmth, dispels cold, expels wind, and eliminates dampness; Agastache rugosa features a fragrant flavor and eliminates dampness, regulates qi, harmonizes the body, and relieves external symptoms; dried tangerine peel promotes the circulation of qi, calms negative energy, and dissipates phlegm; licorice regulates all the herbal components. As an ancient TCM prescription, JMST possesses antitussive, expectorant, spasmolytic, antiallergic, and anti-inflammatory effects. This prescription is used for treating cough and asthma caused by lung heat. Furthermore, it dispels evil using flavorful ingredients with cooling properties, removes lung heat, and relieves asthma. JMST is widely used for treating pneumonia induced by various factors and has achieved favorable curative effects (15–17), and it is also commonly known as “Pneumonia Mixture II” (18). Therefore, JMST is an appropriate remedy for COVID-19 since it is especially effective for pathogenic heat accumulation in the lungs.

According to the climate characteristics and the physique of the local population in Hangzhou, our hospital formulated an “epidemic prevention TCM prescription” for the general adult population. The prescription consisted of 15 g astragalus, 12 g stir-fried ovate atractylodes root, 12 g Poria cocos, 10 g Perillae folium, 9 g wrinkled giant hyssop herb, 9 g honeysuckle flower, 6 g dried tangerine peel, 6 g Saposhnikovia divaricata root, and 3 g licorice. In the concoction, the astragalus features sweet and warm flavors and contributes to the qi of the spleen and the lungs to consolidate superficial defense; stir-fried ovate atractylodes root strengthens the spleen, replenishes qi, and disperses evil; honeysuckle flower features cool and light properties, clears away heat and toxic materials, and dispels wind and heat; Perillae folium relieves external symptoms, dispels cold, and regulates qi; Poria cocos invigorates the spleen and dispels dampness; Agastache rugosa is a fragrant herb that eliminates dampness and regulates qi; dried tangerine peel invigorates the spleen and regulates qi and dryness (19, 20). The first-line clinical patients in our hospital mainly presented phlegmatic hygrosis, so the combined administration of three prescriptions eliminated dampness and filth in patients with the cold-damp constraint or damp-heat accumulation in their lungs. Licorice benefits the throat by clearing away heat and toxic materials, and it also regulates the other herbal components. This prescription tonifies qi, strengthens the exterior, prevents evil spirits, and eliminates turbidity. It also boosts the immunity of the body and prevents infection. At the critical moment of epidemic prevention and control, symptomatic treatments combining TCM and western medicine and the policy of isolation and flow control can achieve satisfactory results (21, 22), confirming the effectiveness of TCM.

During the design of this study, we adopted a therapeutic regimen for the control group according to recommendations from authoritative departments. Additionally, an integrated therapy combining the recommended regimen and JMST was applied in the comprehensive treatment group. Due to the potential risks of COVID-19, we did not create a JMST-only treatment group, which may have led to some deficiencies in the study design. However, results suggested that the combined treatment group achieved better outcomes than the conventional treatment group in terms of the time to obtain a negative test result for nasal/throat swab-based nucleic acid detection, alleviation of clinical symptoms, and absorption time of lung exudation. This indicated that JMST alleviates symptoms, promotes rehabilitation, and reduces sequelae. Therefore, it is necessary to provide a thorough summary and conduct further investigations. The LOS and lymphocyte recovery times in the combined treatment group were also shorter than in the conventional treatment group, but the difference was not statistically significant. This may be related to the exaggerated effect of statistical false negatives due to the small sample size. Besides, there were no significant differences in gastrointestinal reaction, hepatic injury, renal impairment, myocardial injury, and other adverse reactions between the two groups. This indicates that JMST does not aggravate side effects during treatment, which is consistent with the low level of side effects that TCM generally causes. In the treatment of COVID-19 patients, the combined therapy, which integrated authoritative diagnosis and treatment with JMST, alleviated COVID-19 symptoms and promoted the recovery of patients without causing any significant adverse reactions. Thus, JMST achieves favorable therapeutic effects as adjuvant therapy in the treatment of patients with ordinary COVID-19.

It is worth noting that in the third nucleic acid detection sampling, one patient's respiratory tract specimen was single gene positive. This was probably due to the sampling method, conduct of the sampling personnel, or sensitivity of the test reagent. The nucleic acid test result of this patient was negative in the fourth detection test. Moreover, the fifth nucleic acid test returned a negative result and the fecal sample also tested negative. As a result, this patient was confirmed to have a negative nucleic acid test result and was subsequently discharged from the hospital. Few COVID-19 patients were admitted to our hospital during the pandemic in Hangzhou, and the observation durations were relatively short. Also, to avoid further infection, only some patients received the tongue diagnosis and pulse diagnosis. Therefore, in subsequent studies, it is necessary to determine a more suitable population and endpoint index. Furthermore, we must fully consider the feasibility of the sample size and other aspects of the test. Since this study was performed in a single center with a small sample size, there may be some limitations to our results. Therefore, it is necessary to comprehensively explore the effects of JMST on COVID-19 based on a larger sample size. Additionally, the dose of JMST proposed in the *Recommended Scheme for Prevention and Treatment of COVID-19 by Traditional Chinese Medicine (Trial Version 4)* was adopted in this study, and related cytokines were not detected. For that reason, the JMST dosage was not adjusted after patients suffered cytokine storms. Further investigations from this perspective are required to address this limitation of the study.

## Conclusion

In summary, we explored the efficacy of JMST in the treatment of COVID-19 patients based on authoritative diagnosis and treatment schemes after strict statistical analysis. The combined treatment accelerates the alleviation of primary symptoms, promotes the absorption of lung infection lesions, and shortens the time required to obtain a negative nasal/throat swab-based nucleic acid detection result. However, it is still necessary to further investigate shorter LOS and lymphocyte recovery duration based on larger sample sizes. Furthermore, since JMST does not increase the occurrence of adverse reactions, this treatment approach significantly enhances the rehabilitation of COVID-19 patients.

## Data availability statement

The original contributions presented in the study are included in the article/supplementary material, further inquiries can be directed to the corresponding author/s.

## Ethics statement

The studies involving human participants were reviewed and approved by Linping Campus, The Second Affiliated Hospital of Zhejiang University School of Medicine (No. 2019001). Written informed consent for participation was not required for this study in accordance with the national legislation and the institutional requirements. Written informed consent was not obtained from the individual(s) for the publication of any potentially identifiable images or data included in this article.

## Author contributions

JW and X-QZ: conceptualization and research design. FT: data acquisition. SF: data analysis and interpretation. JW and Z-LF: writing of the manuscript. JW and SF: critical revision of the manuscript for intellectual content. All authors have read and approved the final draft.

## Conflict of interest

The authors declare that the research was conducted in the absence of any commercial or financial relationships that could be construed as a potential conflict of interest.

## Publisher's note

All claims expressed in this article are solely those of the authors and do not necessarily represent those of their affiliated organizations, or those of the publisher, the editors and the reviewers. Any product that may be evaluated in this article, or claim that may be made by its manufacturer, is not guaranteed or endorsed by the publisher.
